# Physical Activity of Children and Adolescents with Hearing Impairments: A Systematic Review

**DOI:** 10.3390/ijerph17124575

**Published:** 2020-06-25

**Authors:** Wenhong Xu, Chunxiao Li, Lijuan Wang

**Affiliations:** 1 School of Physical Education and Training, Shanghai University of Sport, Shanghai 200438, China; xuwenhong@sus.edu.cn; 2 School of Physical Education and Sports Science, South China Normal University, Guangzhou 510006, China; cxlilee@outlook.com

**Keywords:** youth, hard of hearing, physical exercise, research synthesis

## Abstract

Physical activity (PA) is important for the development of children and adolescents with hearing impairments (HI). This systematic review aims to summarise the existing literature pertaining to the PA of children and adolescents with HI. A systematic search was conducted on eight major electronic databases. Two reviewers independently screened and selected the returned articles, performed data extraction, assessed methodological quality and synthesised the data using an inductive approach. A total of 15 articles consisting of 14 survey studies and one single-subject intervention study met the inclusion criteria. These studies had good to excellent methodological quality. Participants with HI showed lower levels of participation in PA than participants without disabilities, but they were more physically active than those with other types of disabilities. Amongst the 12 PA correlates identified (i.e., gender, age, mother’s education and social cognitive constructs), only gender was a relatively consistent determinant, and boys are significantly more physically active than girls. Additional studies are needed to confirm the determinants of the PA in children and adolescents with HI to provide strong evidence for the development and implementation of PA interventions for this target group.

## 1. Introduction

Regular participation in physical activity (PA) has physical, psychological, and social benefits for children and adolescents [1,2]. Participation in PA can improve physiological and physical health, such as the improvement of cardiovascular and musculoskeletal health [3], maintenance of healthy weight [4], increase in self-esteem [5] and reduction in anxiety and stress [6]. Moreover, participation in PA is associated with greater social integration among children and adolescents, such as building friendships and enhancing social skills [7,8]. These benefits are particularly important for children and adolescents with disabilities [9]. According to the USA Centers for Disease Control and Prevention [10], developing an overall physically active lifestyle at an early age may decrease one’s chances of developing health-related problems. Moreover, Health People 2020 reaffirmed the importance of PA and identifies “Disability and Health” as one of the topic areas that requires further investigation [11,12].

The World Health Organization (WHO) recommended that children and adolescents with and without disabilities should engage in at least 60 min of moderate-to-vigorous physical activity (MVPA) each day [13]. However, the presence of disability and associated conditions can limit one’s PA participation [14,15]. Indeed, a systematic review recently reported that youth with disabilities were less active than their counterparts without disabilities due to disability-related limitations [16]. These limitations vary across different disability conditions and may result in different levels of PA [17]. Thus, there is a need to learn about the PA participation of youths with a specific disability type, such as hearing impairment (HI).

HI refers to both the complete and partial loss of the ability to hear [18]. Certain studies [19,20] have been conducted to understand the PA levels of children and adolescents with HI and to identify the correlates of their PA participation [17,21]. However, to date, no researchers have reviewed the studies on the PA of children and adolescents with HI. Therefore, the purpose of this systematic review is to summarise and analyse published research literature which described PA participation and examined its influencing factors of children and adolescents with HI. Two specific research questions were focused on: (1) what is the PA level of children and adolescents with HI, and (2) what are factors related to their PA levels? The findings of this review are important for health professionals, teachers and policy makers for developing interventions to increase the PA participation for this study group. Moreover, research gaps in the literature are identified and subsequently offer guidance for future research in this area.

## 2. Methods

This study was conducted in accordance with the Preferred Reporting Items for Systematic Reviews and Meta-Analyses Guideline [22].

### 2.1. Search Strategy

The researchers systematically searched the studies with the following databases from inception to April 2020: Academic Search Premier, Education Resources Information Centre, Education Source, PsycINFO, MEDLINE, SPORTDiscus, Scopus and Web of Science Core Collection. The search strategy included three groups of keywords: (1) hearing impairment^*^ OR hearing disability^*^ OR deaf OR deafness; (2) physical education OR physical activit^*^ OR PA OR MVPA OR exercise^*^ OR health behaviour OR motor activit^*^ OR sport; and (3) young OR youth^*^ OR youngster^*^ OR adolescent^*^ OR teenager OR child^*^ OR childhood OR student^*^ OR pupil^*^. Additionally, the snowballing technique was used to identify potential studies by scanning the references of all the included articles.

### 2.2. Inclusion and Exclusion Criteria

Studies meeting the following criteria were included in this review: (1) empirical studies focusing on the PA levels and factors associated with PA among children and adolescents with HI; (2) studies targeting children with HI (the sample had a mean age below 18 years); and (3) studies were published in English peer-reviewed journals due to language barriers and resource limitations. Studies were excluded if they (1) were pertaining to other topics; (2) did not recruit participants with HI or participants’ mean age was above 18, and (3) were unpublished articles, comments, conference proceedings, and dissertations. The first and third authors independently screened the returned articles according to the inclusion and exclusion criteria. The second author was consulted to resolve the disagreement.

### 2.3. Quality Assessment

To determine the methodological quality of the included studies, the researchers used the adapted McMaster Critical Review Form-Quantitative Studies [23,24]. The form was chosen because it demonstrated good inter-rater agreement of 75–86% [25] and has been used to assess the methodological quality of studies in similar areas [26,27]. The form contains 16 items, which addressed the study purpose (1 item), study background (1 item), study design (1 item), sampling (2 items), measurement (4 items), data analysis (4 items), conclusions (1 item) and implications and limitations (2 item). The researchers scored all items by the degree to which specific criteria were met (yes = 1, no = 0, not applicable = NA). The researchers calculated the summary score for each study by summing the total score obtained across relevant items and dividing it by the total possible score. Scores of ≤50%, 51−75% and >75% were interpreted as low, good and excellent quality, respectively [24]. The first and second authors independently performed the methodological quality assessment. If consensus could not be reached, agreement was obtained through discussion with the third author.

### 2.4. Data Extraction and Synthesis

The first author completed the data extraction and then the second author verified the data. Discrepancies were resolved through a consensus discussion. The researchers extracted the following information: the first author, year of publication, geographic location and methodological details (i.e., research designs, dependent variables, outcome measurements and participant characteristics).

Given the heterogeneity of the included studies, meta-analysis was not conducted. Instead, the researchers used a qualitative synthesis (inductive approach). This study employed the following steps to conduct the qualitative synthesis: (1) read, re-read and reviewed each article to become familiar with the content and context; (2) identified the codes or meaning of units on the basis of the content and context; (3) gathered similar codes to form sub-themes and themes; and (4) revisited themes and combined them under three dimensions (i.e., description of PA levels, comparison of PA levels, and key factors related to PA) [25]. The first and second authors conducted the qualitative data synthesis with on-going consultation, as required, with the third author.

## 3. Results

### 3.1. Search Results

**Figure 1:**
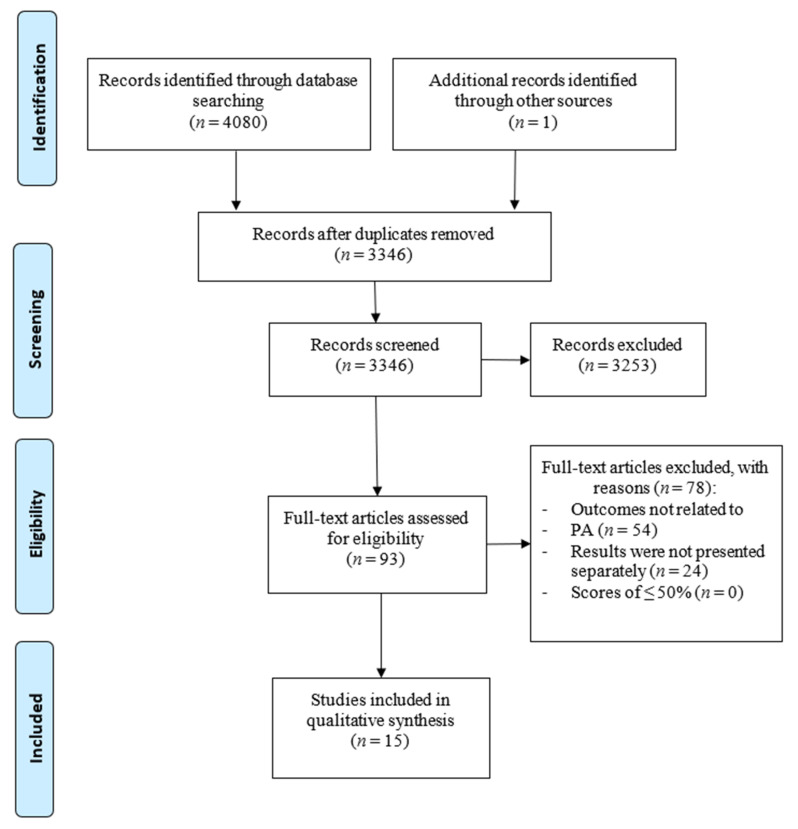
Study selection process. PA—physical activity.

The initial search identified 4080 studies. One additional study was identified by checking the reference lists of the included studies. These studies were exported to EndNote X2, and duplicates were eliminated. The remaining 3346 articles were then subject to screening on the basis of the title and abstract, resulting in the exclusion of 3253 studies. The researchers read the full text of the remaining 93 articles and excluded another 78. Finally, this review included 15 quantitative studies and no qualitative studies met the inclusion criteria (see Figure 1).

### 3.2. Methodological Quality

Table 1 outlines the results of the assessment of methodological quality. Overall, nine out of the 15 included studies (60.0%) were categorised as excellent methodological quality, and the remaining six (40.0%) studies were of good quality. The weakest component amongst the included studies was related to sampling. Specifically, eight studies (53.3%) did not describe the sample in detail and the sample size was not justified in six studies (40.0%).

### 3.3. Study Characteristics

Table 2 presents the study characteristics. The included studies were published between 1991 and 2019, and nine of which (60.0%) were published after 2010. More than half of the studies were conducted either in Hong Kong (*n* = 5, 33.3%) or the USA (*n* = 3, 20.0%). All used a cross-sectional design (*n* = 14, 93.3%) with the exception of one single-subject intervention study (6.7%). The samples included children and adolescents (age range: 3 to 22 years) with mild to severe HI. The sample size ranged from 16 to 6410. The most studied dependent variable was MVPA (*n* = 8, 33.3%), followed by sedentary time (*n* = 4, 16.7%). Eight studies (53.3%) adopted the objective measures of PA, including accelerometers, pedometers and observational tools, whereas seven studies (46.7%) used questionnaires to measure PA.

### 3.4. Major Findings

Three dimensions emerged based on our qualitative synthesis: (a) description of PA levels, (b) comparison of PA levels, and (c) key factors related to PA (see Table 3).

#### 3.4.1. Description of PA Levels

Three studies measured and reported the PA levels of children and adolescents with HI. Two studies by Ng et al. [28] and Lobenius-Palmér et al. [29] used accelerometers to measure the PA of children and adolescents with HI in Finland and Sweden and found the participants engaged in 118 and 110 min/day of MVPA, respectively. However, Li et al. [19] found that children and adolescents with HI in China spent 25 min/day participating in MVPA based on a self-report form.

#### 3.4.2. Comparison of PA Levels

Three themes emerged across 10 included studies, including comparison with students without disabilities (*n* = 5), comparison with other disability types (*n* = 5) and comparison amongst different segments (*n* = 2). Five studies compared PA levels between youths with HI and youths without disabilities and achieved inconsistent findings. Three studies found that students with HI spent less time participating in daily PA than those without disabilities [19,29,30]. By contrast, Ng et al. [28] reported that young adolescents with HI participated more in MVPA and light PA (LPA) per day than those without functional limitations. The study by Williams et al. [20] revealed that no significant difference existed in the MVPA level between these two groups of students.

Five studies [17,29,31,32,33] compared the PA engagement between children and adolescents with HI and those with one of the seven disability types (i.e., visual impairment, physical disabilities, intellectual disabilities, autism spectrum disorders, chronic medical conditions, maladjustment and social development problems). The findings consistently show that youths with HI were more physically active than those with other types of disabilities. Sit et al. [12,32] compared the PA levels of children with HI amongst different segments in school settings, including physical education (PE), recess and lunchtime. Both studies showed that children with HI engaged in more MVPA in recess than during PE or lunchtime.

#### 3.4.3. Key Factors Related to PA

A total of 12 factors associated with the PA of children and adolescents with HI were identified from the 12 included studies in this review. These factors were classified into personal, parental, instructional, psychological and environmental factors.

*Personal factors*: Twelve studies identified personal factors related to the PA participation in children and adolescents with HI. Four subthemes emerged, namely, gender, age, socio-economic level and hearing problems. Six studies examined gender differences in the PA engagement of youths with HI [17,19,29,31,33,34]. Of these studies, five found that boys were more physically active than girls [19,29,31,33,34], and only one study reported no gender difference [17].

Four studies examined age as a personal factor associated with PA and the findings were inconsistent [17,19,29,30]. Engel-Yeger et al. [30] reported a positive association between age and higher levels of PA and intensity, while Lobenius-Palmér et al. [29] showed an opposite finding. The other two studies [17,19] indicated that no significant relationship existed between age and the PA level. Socio-economic level and HI severity were examined by two studies [20,30], which showed that socio-economic level and HI severity were not related to self-reported PA and objectively measured MVPA.

*Parental factors*: Two studies investigated the association between parent-specific factors and the PA participation of their children and adolescents with HI. Engel-Yeger et al. [30] found that the more educated mothers were, the higher the activity intensity level reached by their deaf children in recreational activities. Ellis et al. [35] examined the relationship between four parental factors (i.e., hearing status, participation in deaf sport, values towards physical fitness of their deaf children and values towards sports participation for their deaf children) and the PA of their deaf children. They reported a positive relationship between these four parental factors and their deaf children’s PA participation.

*Instructional factors:* One study provided evidence for the instructional influence on the PA participation of children and adolescents with HI. Through employing a single-subject delayed multiple baseline design, Lieberman et al. [21] examined the effect of peer tutoring on the MVPA time of eight deaf students in inclusive elementary PE classes. The results reveal that after the 11–14 sessions of peer tutoring intervention, deaf students increased their MVPA from 22% to 41.5%.

*Psychological factors:* Three studies addressed psychological factors related to the PA participation of children and adolescents with HI. One study [34] used social-cognitive theory to predict leisure-time PA of 64 children with HI in the USA and Czech Republic. Social cognitive constructs, including barrier self-efficacy, social support from parents, classmates, friends and siblings, PA enjoyment and PE enjoyment, were examined in the study. However, no significant relationship between each of these constructs and leisure-time PA was found. Tsai et al. [36] examined perceived constraints to leisure-time PA participation amongst 149 children and adolescents with HI. An ‘uneasy feeling’ about the attitude of people in mainstream society towards people with disabilities was identified as the most important constraint. Similarly, Li et al. [19] showed that perceived social distance negatively predicted PA participation in 98 adolescents with HI.

*Environmental factors:* Two environmental factors related to the PA participation of children and adolescents with HI were reported in two cross-sectional studies [36,37]. The lack of accessible information, such as how and where to participate, was identified as an important barrier to engagement in PA by students with HI [36]. Seasonal variation was reported as another environmental factor correlated with the MVPA of children with HI. Specifically, children with HI were more physically active in winter than in summer [37].

## 4. Discussion

The purpose of this study was to review the published research literature on PA for children and adolescents with HI. This study included 15 studies, 14 of which employed a cross-sectional survey. Our qualitative synthesis led to three key dimensions, which were the description of PA levels, comparison of PA levels, and key factors related to PA. The discussion will revolve around these three dimensions.

### 4.1. Description of PA Levels

Three studies reported the MVPA time of children and adolescents with HI, and inconsistent findings were achieved. Two studies reported that children and adolescents with HI in Sweden and Finland spent more than 100 min of MVPA per day [28,29], but one study found that children and adolescents with HI in China engaged in only 25 min of MVPA per day [19]. The inconsistent findings may be related to the differences in measures utilized (accelerometers vs. self-report) and culture under which the studies were conducted (European vs. Asian). Although two of the three studies showed that children and adolescents reached the 60 min/day of MVPA recommendation by WHO [13], it is hard to draw a conclusion on the PA level of children and adolescents with HI due to the small number of studies and countries included. Additional studies are needed to report PA level in other countries to understand the global average PA level of children and adolescents with HI.

### 4.2. Comparison of PA Levels

The majority of the studies (three out of five studies) revealed lower levels of participation in PA amongst children and adolescents with HI than those without disabilities. This finding expanded, as well as affirmed, previous reviews, in which a lower level of PA participation amongst children with disabilities than their peers without disabilities was identified [16,38]. This finding is understandable, at least, through three perspectives. Firstly, participation in some forms of PA, such as team sports, dancing and games depends heavily on sensory input and communication skills, which are known to be challenging for children and adolescents with HI [30,39]. Secondly, many students with HI, especially those who are educated in special schools, are uneasy about the attitude of others without disabilities towards them [36]. This social barrier may also inhibit them from participating in PA [19]. Finally, a possible lack of parental support may be another reason contributing to their lower PA level. Parents may restrict their children with HI to participate in out of school PA because of the communication and social interaction issues that accompany HI [28,40].

Interestingly, consistent findings from five studies reveal that children and adolescents with HI were more physically active than other types of disabilities. Although hearing problems are an inhibitor to participate in PA, researchers suggested that they are not a key barrier compared with other disability types, such as physical disability, visual impairment and intellectual disabilities [20]. Moreover, individuals with HI seem to be the same as others without disabilities from physical appearance and the sign of HI is not obvious compared with other disability types. Therefore, those with HI may receive less discrimination from others [31]. This may be another reason for more frequent PA participation amongst this group than other disability groups.

Two studies compared the PA level of children and adolescents with HI amongst different school segments, including PE class, recess, and lunchtime. Both studies showed that children and adolescents with HI were more physically active in recess than during PE class and lunchtime. This finding is somewhat unexpected because many studies on students without disabilities found that PE contributed most in their PA participation [41,42,43]. PE is structured and requires time to be allocated to management and instruction to achieve desirable learning outcomes. However, it tends to take a longer time to manage and instruct students with disabilities (i.e., students with HI) than those without disabilities in PE [44]. As such, the PA participation time of students with disabilities is compromised [32,45]. By contrast, recess is non-structured and students are free to move and choose activities, which may contribute to greater PA participation.

### 4.3. Key Factors Related to PA

When identifying key PA correlates of children and adolescents with HI, five subthemes and 12 factors emerged. Amongst these 12 factors, two personal factors, including gender and age, were the most studied ones, which were investigated in six and four studies, respectively. Five out of six studies supported the idea that boys were significantly more physically active than girls. This finding is consistent with previous reviews on the PA of youth with disabilities [46] and without disabilities [47,48]. Despite the physiological deficit, gender difference is similar between children with and without HI. Bravery, aggressiveness and perseverance are valued in boys, whereas gentleness, kindness, approachability, sensitivity, quietness, weakness and malleability are valued in girls [49]. These gender differences may explain why boys are more active than girls, suggesting the girls’ group should be targeted to increase PA levels. With regard to the age difference, a definite conclusion cannot be drawn because of inconsistent findings amongst the four studies. The inconsistency may be attributed to the varying study characteristics that were conducted in four different countries, including participant characteristics, education systems and social cultures. More studies are therefore needed to examine age difference to confirm this relationship.

It is notable that the relationship between each of the other ten factors and PA was investigated by only one study. Moreover, only one interventional study targeted the PA participation of children and adolescents with HI. Children and adolescents with HI have severe reading comprehension problems, which could increase difficulties in conducting research with them [50]. Based on these existing studies, drawing a conclusion on the association between these factors and the PA participation of children and adolescents with HI is difficult. Additional studies are needed to confirm the relationship between these factors and the PA in this target group.

## 5. Limitations and Implications for Future Research

Several limitations inherent within the current review should be noted. Firstly, although this study conducted an extensive literature search on eight major databases to identify potential studies, a few published studies were possibly missed in this review because our search was limited to English journal articles. Second, this review included only one intervention study, and conclusions were largely drawn on the basis of the cross-sectional evidence. Therefore, inferring causation was difficult. Finally, given the small number of studies included and their heterogeneity (i.e., participant characteristics and PA measures), a meta-analysis could not be conducted.

Despite the outlined limitations, the findings of the present review can shed some light on future research directions and practical implications. Firstly, most included studies were from English-speaking countries in North America and Europe. Language barriers may be the reason for the lack of studies from other countries. The literature search showed that studies had been published in Asian or Arabian countries in their own language, but they were not included in this review because of the language barriers and resource limitations. Secondly, most of the included studies used a cross-sectional research design. Although cross-sectional studies may generalise the research finding to the whole group of children and adolescents with HI, too few intervention studies targeting children and adolescents with HI limited the promotion of their PA participation, especially as this group of youths was reported to be physically inactive [19,29,30]. Meanwhile, qualitative data are also needed to obtain in-depth information about the thoughts and feelings of students with disabilities on their PA participation [51].

## 6. Conclusions

This systematic review provides some information for understanding PA levels and their correlates amongst children and adolescents with HI. Generally, studies consistently found a lower level of PA participation amongst children and adolescents with HI than those without disabilities. However, they were more physically active than those with other types of disabilities. Amongst the 12 factors identified, only gender was consistently reported to be associated with PA, and boys are more physically active than girls. To further our understanding, more studies are needed to report PA levels of children and adolescents with HI from various countries and examine factors related to PA and to provide evidence for the development and implementation of PA interventions for individuals with HI.

## Figures and Tables

**Table 1:** Results of study quality evaluation using the adapted McMaster Critical Review Form for Quantitative Studies.

Study	Items																Score		Quality
	Q1	Q2	Q3	Q4	Q5	Q6	Q7	Q8	Q9	Q10	Q11	Q12	Q13	Q14	Q15	Q16	Raw	%	
Sit et al. (2017) [12]	1	1	1	0	1	1	1	1	1	1	1	1	1	1	1	1	15	93.8	Excellent
Longmuir et al. (2000) [17]	1	1	1	1	1	1	1	1	1	1	1	1	1	1	0	1	15	93.8	Excellent
Li et al. (2018) [19]	1	1	1	0	0	1	1	1	1	1	1	1	1	1	1	1	14	87.5	Excellent
Williams et al. (2017) [20]	1	1	1	0	1	1	1	1	1	1	1	0	1	1	1	1	14	87.5	Excellent
Lieberman et al. (2000) [21]	1	1	1	1	0	0	1	1	1	0	1	0	1	1	1	1	12	75.0	Good
Ng et al. (2019) [28]	1	1	1	1	0	1	0	0	1	1	1	1	1	1	0	1	12	75.0	Good
Lobenius-Palmér et al. (2018) [29]	1	1	1	1	1	1	1	1	1	1	1	1	1	1	1	1	16	100.0	Excellent
Engel-Yeger et al. (2013) [30]	1	1	1	0	0	1	1	1	1	1	1	1	1	0	0	1	12	75.0	Good
Sit et al. (2002) [31]	1	1	1	1	1	1	1	1	1	1	1	1	1	1	1	0	15	93.8	Excellent
Sit et al. (2007) [32]	1	1	1	1	1	1	1	1	1	1	1	1	1	1	1	0	15	93.8	Excellent
Suzuki et al. (1991) [33]	1	0	1	1	1	1	0	0	1	1	1	0	1	1	1	0	11	68.8	Good
Martin et al. (2013) [34]	1	1	1	0	0	1	1	1	1	0	1	0	1	1	1	1	12	75.0	Good
Ellis et al. (2013) [35]	1	1	1	0	1	1	1	1	1	1	1	1	1	1	1	0	14	87.5	Excellent
Tsai et al. (2005) [36]	1	1	1	0	0	0	1	1	1	1	1	0	1	1	1	1	12	75.0	Good
Sit et al. (2019) [37]	1	1	1	0	1	1	1	1	1	1	1	1	1	1	1	1	15	93.8	Excellent

Note. Q1: Was the study purpose stated clearly?; Q2: Was the relevant background literature reviewed?; Q3: Was the design appropriate for the research question?; Q4: Was the sample described in detail?; Q5: Was the sample size justified?; Q6: Was informed consent obtained?; Q7: Were the outcome measures reliable?; Q8: Were the outcome measures valid?; Q9: Was the method described in detail; Q10: Were results reported in terms of statistical significance?; Q11: Were the analysis methods appropriate?; Q12: Was importance for the practice reported?; Q13: Were any drop-outs reported?; Q14: Were the conclusions appropriate given the study methods?; Q15: Are there any implications for practice given the results of the study?; Q16: Were limitations of the study acknowledged and described by the authors? 1: Yes, 0: No.

**Table 2:** Study characteristics of included studies.

First Author (Year)	Geographic Location	Research Design	Sample Characteristics	HI Level	Dependent Variables	Measures
Sit (2017) [12]	Hong Kong	Cross-sectional	259 children (12 with HI) Gender: male (154), female (105) Age and/or grade: M = 13.04, SD = 4.45	HI	MVPA and sedentary time	Accelerometers
Longmuir (2000) [17]	Canada	Cross-sectional	957 youths (164 with HI) Gender: male (499), female (458) Age and/or grade: 6–20 years	Deaf; hard of hearing	Habitual PA	Modified version of Canada Fitness Survey
Li (2018) [19]	China	Cross-sectional	197 students (98 were deaf) Gender: male (85), female (112) Age and/or grade: M = 15.73, SD = 1.50	Deaf	LPA, MVPA, and MET	IPAQ-SF
Williams (2017) [20]	UK	Cross-sectional	6410 children (745 with HI) Gender: male (48.9%), female (51.1%) Age and grade: mean 7.2 (SD =2.4)	HI	MVPA and sedentary time	Accelerometers
Lieberman (2000) [21]	USA	Single-subject intervention	8 deaf students and 8 hearing peers Gender: male (8), female (8) Age and/or grade: 10–12 years, grade 4–6	Deaf	MVPA	SOFIT
Ng (2019) [28]	Finland	Cross-sectional	1436 adolescents (15 with HI) Gender: male (571), female (865) Age and/or grade: 11–15 yeas	HI	LPA and MVPA	Accelerometers
Lobenius-Palmér (2018) [29]	Sweden	Cross-sectional	102 youths with disabilities (19 with HI) and 800 youths with typical development Gender: male (59), female (43) Age and/or grade: 7–20 years	HI and deafness	Average PA, LPA, MVPA, and sedentary time	Accelerometers
Engel-Yeger (2013) [30]	Israel	Cross-sectional	70 children (25 with HI) Gender: male (39), female (31) Age and/or grade: 6–11 years	2 moderate hearing loss; 23 severe-profound hearing loss	Leisure-time PA	CAPE
Sit (2002) [31]	Hong Kong	Cross-sectional	237 children (41 with HI) Gender: male (143), female (94) Age and/or grade: 9–19 years	HI	Daily PA	Questionnaires
Sit (2007) [32]	Hong Kong	Cross-sectional	172 children (16 with HI) Gender: NA Age and/or grade: Grade 4–6	Hearing impairment	MVPA	SOFIT
Suzuki (1991) [33]	Japan	Cross-sectional	2222 students (346 were deaf) Gender: male (1384), female (838) Age and/or grade: 3–22 years	Deaf	Daily PA	Pedometers
Martin (2013) [34]	Czech Republic and USA	Cross-sectional	64 children with HI Gender: male (42), female (22) Age and grade: M = 14.1, SD = 2.1	HI	Leisure-time PA	GLTEQ
Ellis (2013) [35]	USA	Cross-sectional	128 deaf children and their parents Gender: male (73), female (55) Age and/or grade: M = 96.38 months, grade 1–4	Deaf	PA habits	Questionnaires
Tsai (2005) [36]	Hong Kong	Cross-sectional	149 students with HI Gender: male (53.7%) and female (46.3%) Age and/or grade: 12–20 years	Severe and profound hearing loss	Leisure-time PA	Questionnaires
Sit (2019) [37]	Hong Kong	Cross-sectional	270 children (11 with HI) Gender: male (162), female (108) Age and/or grade: Grade 1–12	HI	MVPA and sedentary time	Accelerometers

HI: hearing impairment; PA: physical activity; LPA: light physical activity; MVPA: moderate to vigorous physical activity; MET: Metabolic Equivalent Task; SOFIT: System for Observing Fitness Instruction Time; CAPE: Children’s Assessment of Participation and Enjoyment; GLTEQ: Godin Leisure-time Exercise Questionnaire; IPAQ-SF: International Physical Activity Questionnaire-Short Form; NA: not applicable.

**Table 3:** Summary of the key themes and findings.

Dimensions	Themes	Sub-Themes	PA Levels/Differences/Associations	Studies
PA levels (*n* = 3)	MVPA time	NA	118 min/day of MVPA	Ng et al., 2019 [28]
			25 min/day of MVPA	Li et al., 2018 [19]
			111 min/day of MVPA	Lobenius-Palmér et al., 2018 [29]
Comparison of PA levels (*n* = 10)	Comparison with students without disabilities (*n* = 5)	NA	Lower PA level in the group with HI	Li et al., 2018 [19]; Lobenius-Palmér et al., 2018 [29]; Engel-Yeger et al., 2013 [30]
			Higher PA level in the group with HI	Ng et al., 2019 [28]
			No significant difference in PA level	Williams et al., 2017 [20]
	Comparison with students with other disability types (*n* = 5)	NA	Higher PA level in the group with HI than other disability groups	Longmuir et al., 2000 [17]; Lobenius-Palmér et al., 2018 [29]; Sit et al., 2002 [31]; Sit et al., 2007 [32]; Suzuki et al., 1991 [33]
	Comparison among different segments (*n* = 2)	Comparison during PE lessons and recess (*n* = 1)	More active at recess	Sit et al., 2007 [32]
		Comparison among PE lessons, recess, and lunch time (*n* = 1)	More active at recess	Sit et al., 2017 [12]
Key factors related to PA (*n* = 12)	Personal factors (*n* = 12)	Gender (*n* = 6)	Males were more physically active than girls	Li et al., 2018 [19]; Lobenius-Palmér et al., 2018 [29]; Sit et al., 2002 [31]; Suzuki et al., 1991 [33]; Martin et al., 2013 [34]
			No significant gender differences	Longmuir et al., 2000 [17]
		Age (*n* = 4)	Positively related to the activity level of children with HI	Engel-Yeger et al., 2013 [30]
			Negatively related to the MVPA level of youth with HI	Lobenius-Palmér et al., 2018 [29]
			Not related to activity level of youth with HI	Longmuir et al., 2000 [17]; Li et al., 2018 [19]
		Socio-economic level (*n* = 1)	Not related to the activity level of children with HI	Engel-Yeger et al., 2013 [30]
		Hearing impairments (*n* = 1)	Not related to daily PA level of children with HI	Williams et al., 2017 [20]
	Parental factors (*n* = 2)	Mother’s years of education (*n* = 1)	Positively related to the activity level of their children with HI	Engel-Yeger et al., 2013 [30]
		Parents’ hearing status, participation in Deaf sport, values toward physical fitness and sports participation for their deaf children (*n* = 1)	Positively related to their deaf children’s PA levels	Ellis et al., 2013 [35]
	Instructional factors (*n* = 1)	Peer tutoring (*n* = 1)	Peer tutoring significantly increased PA participation of students with HI	Lieberman et al., 2000 [21]
	Psychological factors (*n* = 3)	Social cognitive constructs (barrier self-efficacy, social support from parents, classmates, friends, and siblings, PA enjoyment, and PE enjoyment) (*n* = 1)	No significant relationship between each of these constructs and leisure-time PA of children with HI	Martin et al., 2013 [34]
		“Uneasy feeling” about attitude of the mainstream society (*n* = 1)	An important constraint of the leisure-time PA participation of children	Tsai et al., 2005 [36]
		Social distance (*n* = 1)	Negatively related to PA participation in adolescents with HI	Li et al., 2018 [19]
	Environmental factors (*n* = 2)	Lack of accessible information (*n* = 1)	An important barrier to the PA involvement of students with HI	Tsai et al., 2005 [36]
		Seasonal variation (*n* = 1)	More physically active in winter than in summer	Sit et al., 2019 [37]

PE: physical education; HI: hearing impairment; PA: physical activity; NA: not applicable.
